# Dietary habits and psychological disorders in a large sample of Iranian adults: a population-based study

**DOI:** 10.1186/s12991-020-00263-w

**Published:** 2020-02-25

**Authors:** Zohreh Sadat Sangsefidi, Elnaz Lorzadeh, Mahdieh Hosseinzadeh, Masoud Mirzaei

**Affiliations:** 1 Nutrition and Food Security Research Center, Shahid Sadoughi University of Medical Sciences, Yazd, Iran; 2 Department of Nutrition, School of Public Health, Shahid Sadoughi University of Medical Sciences, Yazd, Iran; 3 Yazd Cardiovascular Research Centre, Shahid Sadoughi University of Medical Sciences, Yazd, Iran

**Keywords:** Psychological disorders, Dietary habits, Diet

## Abstract

**Background:**

Since an association has been found between diet and psychological problems, this study aimed to evaluate the relationship between dietary habits and psychological problems among Iranian adults.

**Methods:**

Data on dietary habits and psychological problems among 9965 adults were provided from the recruitment phase of Yazd Health Study, a population-based cohort research on Iranian adults. The association between dietary habits and psychological problems was assessed by multiple logistic regression analysis.

**Results:**

After the adjustment for confounders, fast food and fried food consumptions were associated with increased depressive [odds ratio (OR) = 1.61, 95% confidence interval (CI) 1.18–2.20] and stress symptoms (OR = 2.47, 95% CI 1.46–4.18), respectively. Eating breakfast had a protective role on stress features (OR = 0.60, 95% CI 0.39–0.92), while snack intake was related to higher odds of depressive (OR = 1.36, 95% CI 1.01–1.84), anxiety (OR = 1.99, 95% CI 1.55–2.56) and stress symptoms (OR = 1.73, 95% CI 1.23–2.54). There was also an inverse association between sweetened drink consumption and depressive (OR = 0.76, 95% CI 0.59–0.96), anxiety (OR = 0.76, 95% CI 0.62–0.93), and stress features (OR = 0.73, 95% CI 0.55–0.97; OR = 0.63, 95% CI 0.48–0.82).

**Conclusions:**

Even though an inverse relationship was found between sweetened drinks intake and psychological problems, snack consumption was associated with higher chances of them. Eating breakfast had a protective effect on stress symptoms, while fast food and fried food intakes were related to increased depressive and stress features chances, respectively.

## Background

Psychological problems are the most leading causes of ill health such as cardiovascular disease (CVD), stroke, cancer, and general disability around the world [1]. They can adversely associate with health status, quality of life, and ability to work [2]. Depressive, stress, and anxiety symptoms are the most common psychological problems [3]. Depression affects about 121 million people around the world and the prevalence rates of depression and anxiety among Iranian adults are reported to be respectively 21.0% and 20.8% [4].

Although with the unknown precise etiology of psychiatric disorders, several modifying factors, including environmental, genetic, biological, and overall psychological health, might contribute to these conditions [5]. Mood and psychological health are undeniably affected by what we eat, and this is not only true for the developmental period but also throughout adulthood as well [6].

Current evidence suggests that poor psychological health may associate with breakfast skipping and the likeliness of having a less-balanced diet [7]. People diagnosed with depression are more likely to skip breakfast [8]. Furthermore, omitting breakfast as well as breakfast that lacks in core food groups such as cereals, fruit, vegetable, meat, and alternatives and milk may result in psychological distress and poor mindful attention [9, 10]. Multiple studies also found a relationship between stress and higher intake of snack [11–14] and fast food [14, 15]. Similarly, women with depressive symptoms had statistically greater fast food intake compared to women who did not report depressive symptoms [16]. Meanwhile, a significant lower prevalence of depression symptoms was found in individuals with a low consumption of ‘ready-to-eat’ foods [17]. It has been also found that a high level of soft drink consumption was related to increased risk of psychological distress [18–21]. According to a recent research, diets rich in processed or fried foods had a significant relationship with greater risk of psychiatric disorders [22]. Several cross-sectional studies suggest that frequent drinking of sweetened drinks can associate with higher prevalence of depression and stress [20, 23, 24]. In some of these studies, mental health indicators and food consumption assessment were based on self-report and could possibly be misdirected to different social desirability. Lack of generalization to a larger population was also another limitation in studies along with the discrepancies and limited survey tool to collect psychological data [11, 13, 25]. Furthermore, most of the studies evaluating the relationship between dietary habits and psychological problems have been accomplished in western countries which are different in culture and dietary behavior with Mediterranean region countries, including Iran. Few studies have also been performed in Middle East countries in this field. One population-based study in Iran suggested a relationship between dietary behavior and stress, in which participants with low levels of stress consumed a healthier diet (with higher dietary intake of unsaturated oils, grains, fruits, vegetables, meat, and dairy products) than high-stressed people [26]. However, other mental disorders such as depression and anxiety disorders have not been assessed in this survey [26].

Limited studies (specifically population-based ones) regarding to the relationship between dietary habits and psychological problems have been conducted in Mediterranean countries. Furthermore, because of the discrepancies in the prevalence of psychiatric disorders in different countries (Mediterranean countries like Iran and western countries) and inconsistencies between the outcomes of previous studies, this population-based study aimed to investigate the association between dietary habits and psychological problems including depression, anxiety, and stress among a representative large sample of Iranian adults.

## Materials and methods

### Study population and data collection

In the present research, participants were selected from Yazd Health Study (YaHS), which has been conducted since 2014 on a large random sample of Iranian population of Yazd Greater Area aged 20–70 years, aiming to assess the changing incidence of variety of chronic diseases and their associated risk factors.

The recruitment phase was conducted during September 2014–March 2016 based on cluster sampling method. Then, adults of ages 20–70 years (*n* = 10,000) were selected using a two-level clustered random sampling method based on WHO STEP guidelines [27]. Briefly, first, 200 clusters were randomly selected based on city postcodes. Then, the interviewers arranged a meeting time with the residences at the assigned addressees after explaining the study. Finally, the interviewers visited the neighborhood of the first addressee based on the study protocol, to interview 50 participants assigned to each cluster: 25 men and 25 women; there are five subjects in each 10-year categorized age group (20–29, 30–39, 40–49, 50–59, and 60–69 years) [28]. The survey was approved by the Ethics Committee of Shahid Sadoughi University of Medical Sciences, Yazd, Iran (Ethical approval code: IR.SSU.REC.1393.7341, Date: July 8, 2014). Informed consents were also taken from all the participants. A validated questionnaire was provided to collect the data on population characteristics, smoking history, history of chronic disease (including hypertension, diabetes, cardiovascular disease, cancer, depression, and dyslipidemia), physical activity, anthropometric measurements, psychological health status, blood pressure measurement, and biochemical and dietary assessments. A pilot research on 50 participants was also performed before the study, whereby reliability and validity of questionnaires were evaluated by experts of each section. Moreover, the reliability of the questionnaire was confirmed by Cronbach’s alpha 0.89. More details on YaHS database have been recently published and reported [29].

### Dietary assessment

Dietary habits were assessed by asking participants about the frequency or serving consumption of the following items: sweetened drinks (fruit juices, artificially or homemade sweetened beverages) (frequency: not at all, less than once per week, once or more per week), fast food and canned food (frequency: not at all or few times per year, 1–3 times per months, once or more per week), breakfast (frequency: not at all, once per week, more than once per week), sugar cubes (one sugar cube contains almost 3 g sugar) (serving: not at all, 1–2 cubes per day, more than 2 cubes per day), fried food (frequency: not at all, less than once per week, once or more per week), and snack (frequency: not at all, less than once per week, once or more per week).

### Anthropometric measurements

The body weight was measured while standing in the middle of the scale with the minimum possible clothing, using Omron BF511 (Omron Inc. Osaka, Japan) portable digital scale and the body analyzer with the accuracy of 0.1 kg. To measure participant’s height, they were barefoot with their head, shoulder blades, and heels touching the straight wall, to which a tape measure was fixed. Body Mass Index (BMI) was calculated after dividing the body weight (kg) by the square of height (m).

### Psychological health assessment

The Iranian validated version of depression, anxiety, and stress scale questionnaire 21 (DASS 21), a well-known short version of self-report with seven items per subscale [30] was used to screen the psychological problems including depression, anxiety, and stress. After reading the statements, participants were asked to record their immediate response based on a four-point like-type scale ranging from 0 (Does not apply to me at all) to 3 (Applies to me very much or most of the time). Then, the scores were summed for each scale for identified items. Since the DASS 21 is a short version of DASS (42-item self-report questionnaire), the summed up scores were multiplied by two (×2). As a result, depression, anxiety, and stress are defined by the following scores: ≥ 10, ≥ 8, ≥ 15, respectively.

### Physical activity assessment

Physical activity among participants was evaluated using the short form of the International Physical Activity Questionnaire (IPAQ) [31]. The validity of Persian translation for this questionnaire has previously been established by Moghaddam et al. [32]. Eventually, physical activity levels of each individual were classified as low, medium, and high according to guidelines of IPAQ short form [31].

### Statistical analysis

Statistical Package for Social Science (SPSS Inc., Chicago IL. Version 16.0) was used for statistical analysis. To describe qualitative variables, frequency and percentage were used. Moreover, logistic regression analysis was performed to evaluate the relation between dietary habits and psychological problems in different models with the lowest frequency as a reference for all the models and adjustment for various confounding factors as follows: age (20–29, 30–39, 40–49, 50–59, 60–69 years), educational level (secondary school and lower, high school, diploma and graduate diploma, bachelors, masters, and PhD), physical activity (low, medium, and high), history of chronic diseases (yes/no, including: hypertension, diabetes, cardiovascular disease, cancer, depression, and dyslipidemia), smoking history (yes/no), and BMI. Statistical significance was also considered when *p* < 0.05.

## Results

### Characteristics of participants

General characteristics of the study population (*n* = 9965) are recorded in Table 1. Psychological status of the subjects was reported as 8.6% depressive, 13.2% anxiety, and 13.2% stress symptoms in the study population. Table 2 demonstrates dietary habits among participants. As reported, sweetened drinks’ (44.1%), sugar cubes’ (61%), and fried foods’ (71.5%) consumption in majority of the participants were once or more than once a week, whereas other dietary habits were more prominent in less than once per week. Most of the people (%93.2) are also breakfast consumers. Furthermore, assessing physical activity indicated that the majority of the subjects (92.6%) had low (50.8%) or medium (41.8%) physical activity level.

**Table 1:** Distribution of all participants according to general characteristics

Variables	Total (*n* = 9965)
	*N* (%)
Sex	
Male	4921 (49.7%)
Female	4989 (50.3%)
Age (years)	
20–29	1963 (19.8%)
30–39	2025 (20.4%)
40–49	2049 (20.7%)
50–59	1969 (19.9%)
60–69	1907 (19.2%)
Education level	
Secondary school and lower	5389 (54.6%)
Diploma and Graduate diploma	2932 (29.7%)
Bachelors	1291 (13.1%)
Masters and PhD	254 (2.6%)
Smoking	
Yes	1056 (10.9%)
No	8610 (89.1%)
Marital status	
Married	8430 (85%)
Single	1054 (10.6%)
Widowed	380 (3.8%)
Divorced	55 (0.6%)
BMI	
Low weight (< 18.5)	255 (2.7%)
Normal (18.5–24.9)	2944 (31.1%)
Overweight (25–29.9)	3551 (37.5%)
Obesity (≥ 30)	2707 (28.6%)
Psychological status	
Depression status	
Normal	8836 (91.4%)
Depression	827 (8.6%)
Anxiety status	
Normal	8441 (86.8%)
Anxiety	1279 (13.2%)
Stress status	
Normal	9026 (94%)
Stress	1279 (13.2%)

**Table 2:** Distribution of participants according to dietary intakes

Dietary habits^a^	Total (*n* = 9965)
	*N* (%)
Sweetened drinks	
Not at all	2464 (24.7%)
Lower than once per week	3109 (31.2%)
Once or more per week	4392 (44.1%)
Fast foods	
Not at all or few times per year	5997 (60.2%)
1–3 times per month	2899 (29.1%)
Once or more per week	1069 (10.7%)
Canned foods	
Not at all or few times per year	6982 (70.1%)
1–3 times per month	2358 (23.7%)
Once or more per week	625 (6.3%)
Breakfast	
Not at all	395 (4.0%)
Once per week	282 (2.8%)
More than once per week	9288 (93.2%)
Sugar cubes (one sugar cube contains almost 3 g sugar)	
Not at all	1613 (16.2%)
1–2 cubes per day	2270 (22.8%)
More than 2 cubes per day	6082 (61.0%)
Fried foods	
Not at all	646 (6.5%)
Lower than once per week	2191 (22.0%)
Once or more per week	7128 (71.5%)
Snacks	
Not at all	6389 (64.1%)
Lower than once per week	2281 (22.9%)
Once or more per week	1295 (13.0%)

^a^Dietary habits for all items were presented as frequency of consumption except for sugar cubes which presented as serving of intake

### Dietary habits and depressive symptoms

The association between dietary habits and depressive symptoms is presented in Table 3. After adjusting for several confounding factors such as age, education level, physical activity, history of chronic diseases, and smoking, consuming sweetened drinks for once or more per week was significantly related to lower odds of depressive features in oppose to its lack of consumption (OR = 0.78, 95% CI 0.62–0.99). This association did not change after further adjustment for BMI (OR: 0.76, 95% CI 0.59–0.96). Moreover, fast food consumption for once or more per week had significant correlations with depressive symptoms when compared to those who had either no fast foods intake or stated to consume fast foods less than a few times per year (OR: 1.61, 95% CI 1.18–2.18), even after additional adjustment for BMI (OR: 1.61, 95% CI 1.18–2.20). Correspondingly, consumption of snacks for less than once per week (OR: 1.30, 95% CI 1.02–1.65) as well as once or more per week (OR: 1.39, 95% CI 1.03–1.87) had significant influences on depressive features in contrast to those who did not have any consumption at all. However, we observed that participants with snacks consumption of only once or more per week had higher odds ratio for depressive symptoms than of those who did not consume snacks at all, after further adjustment for BMI (OR 1.36, 95% CI 1.01–1.84). It is also worth mentioning that no significant relation was found between other dietary habits and depressive features.

**Table 3:** Multivariable-adjusted odds ratios (95% CI) for depression across different frequencies or servings of dietary habits

Dietary habits^a^	Depression			
	Multivariable adjusted^b^		Multivariable + BMI^c^	
	OR	95% CI	OR	95% CI
Sweetened drinks				
Not at all	Reference		Reference	
Lower than once per week	0.91	0.72–1.16	0.92	0.72–1.18
Once or more per week	0.78*	0.62–0.99	0.76*	0.59–0.96*
Fast foods				
Not at all or few times per year	Reference		Reference	
1–3 times per month	1.25	0.99–1.59	1.24	0.97–1.58
Once or more per week	1.61*	1.18–2.18*	1.61*	1.18–2.203*
Canned foods				
Not at all or few times	Reference		Reference	
1–3 times per months	0.99	0.79–1.23	0.99	0.79–1.24
Once or more per week	1.13	0.79–1.60	1.12	0.78–1.61
Breakfast				
Not at all	Reference		Reference	
Once per week	1.35	0.79–2.37	1.37	0.78–2.40
More than once per week	0.72	0.49–1.07	0.74	0.49–1.11
Sugar cubes (one sugar cube contains almost 3 g sugar)				
Not at all	Reference		Reference	
1–2 cubes per day	0.95	0.71–1.27	0.95	0.70–1.59
More than 2 cubes per day	1.07	0.82–1.39	1.03	0.69–1.52
Fried foods				
Not at all	Reference		Reference	
Lower than once per week	1.17	0.78–1.74	1.06	0.71–1.59
Once or more per week	1.14	0.77–1.68	1.03	0.69–1.52
Snacks				
Not at all	Reference		Reference	
Lower than once per week	1.302*	1.02–1.65*	1.27	0.99–1.63
Once or more per week	1.39*	1.03–1.87*	1.36*	1.01–1.84*

*OR* odds ratio, 95% *CI 95%* confidence interval

*Significance level was considered as *p* < 0.05

^a^Dietary habits for all items were presented as frequency of consumption except for sugar cubes which presented as serving of intake

^b^Adjusted for age (20–29, 30–39, 40–49, 50–59, 60–69 years), education level (secondary school and lower, High school, Diploma and Graduate diploma, Bachelors, Masters, and PhD), physical activity level (low/medium/high), history of chronic diseases (hypertension, diabetes, cardiovascular disease, cancer, depression, and dyslipidemia), smoking (yes/no)

^c^Adjusted for age (20–29, 30–39, 40–49, 50–59, 60–69 years), education level (secondary school and lower, High school, Diploma and Graduate diploma, Bachelors, Masters, and PhD), having physical activity (yes/no), history of chronic diseases (hypertension, diabetes, cardiovascular disease, cancer, depression, and dyslipidemia), smoking (yes/no), and BMI

### Dietary habits and anxiety symptoms

According to Table 4, sweetened drinks’ consumption for once or more per week in contrast with non-consumption of them had a significant effect on anxiety symptoms (OR: 0.77, 95% CI 0.64–0.94). This result was unchanged after further adjustment for BMI (OR: 0.76, 95% CI 0.62–0.93). As for snacks consumption, the significant differences associated with chance of anxiety features were found between none-consumers and those who consumed for less than once (OR 1.64, 95% CI 1.34–2.01), once or more per week (OR: 2.01, 95% CI 1.58–2.56). These findings continue to remain significant after the additional adjustment for BMI [OR (less than once per week: 1.6, 95% CI 1.3–1.96; once or more per week: OR: 1.99, 95% CI 1.55–2.56]. Meanwhile, no significant association of anxiety symptoms with other dietary habits was observed among subjects.

**Table 4:** Multivariable-adjusted odds ratios (95% CI) for anxiety across different frequencies or servings of dietary habits

Dietary habits^a^	Anxiety			
	Multivariable adjusted^b^		Multivariable + BMI^c^	
	OR	95% CI	OR	95% CI
Sweetened drinks				
Not at all	Reference		Reference	
Lower than once per week	1.007	0.82–1.22	1.01	0.83–1.24
Once or more per week	0.77*	0.64–0.94*	0.76*	0.62–0.93*
Fast foods				
Not at all or few times per year	Reference		Reference	
1–3 times per month	1.03	0.85–1.25	1.06	0.86–1.29
Once or more per week	1.15	0.88–1.502	1.19	0.908–1.56
Canned foods				
Not at all or few times	Reference		Reference	
1–3 times per months	1.14	0.96–1.36	1.12	0.94–1.35
Once or more per week	1.11	0.82–1.504	1.13	0.83–1.54
Breakfast				
Not at all	Reference		Reference	
Once per week	1.35	0.84–2.16	1.25	0.77–2.03
More than once per week	0.74	0.53–1.04	0.74	0.53–1.05
Sugar cubes (one sugar cube contains almost 3 g sugar)				
Not at all	Reference		Reference	
1–2 cubes per day	1.11	0.88–1.40	1.08	0.85–1.37
More than 2 cubes per day	1.05	0.85–1.30	1.03	0.82–1.28
Fried foods				
Not at all	Reference		Reference	
Lower than once per week	1.21	0.87–1.66	1.19	0.85–1.65
Once or more per week	1.03	0.75–1.41	1.01	0.73–1.39
Snacks				
Not at all	Reference		Reference	
Lower than once per week	1.64*	1.34–2.01*	1.60*	1.31–1.96*
Once or more per week	2.01*	1.58–2.56*	1.99*	1.55–2.56*

*OR* odds ratio, *95% CI* 95% confidence interval

*Significance level was considered as *p* < 0.05

^a^Dietary habits for all items were presented as frequency of consumption except for sugar cubes which presented as serving of intake

^b^Adjusted for age (20–29, 30–39, 40–49, 50–59, 60–69 years), education level (Secondary school and lower, High school, Diploma and Graduate diploma, Bachelors, Masters, and PhD), physical activity history(yes/no), history of chronic diseases (hypertension, diabetes, cardiovascular disease, cancer, depression and dyslipidemia), and smoking (yes/no)

^c^Adjusted for age (20–29, 30–39, 40–49, 50–59, 60–69 years), education level (Secondary school and lower, High school, Diploma and Graduate diploma, Bachelors, Masters, and PhD), physical activity level (low/medium/high), history of chronic diseases (hypertension, diabetes, cardiovascular disease, cancer, depression, and dyslipidemia), smoking (yes/no), and BMI

### Dietary habits and psychological stress symptoms

Assessing the relation between dietary habits and stress symptoms is reported in Table 5. According to the results, less than once a week (OR: 0.71, 95% CI 0.54–0.94), once or more per week (OR:0.67, 95% CI 0.52–0.87) consumptions of sweetened drinks are associated with lower chances of stress features compared to those who do not consume at all. This association did not change even after further adjustment for BMI (less than once a week: OR: 0.73, 95% CI 0.55–0.97; once or more per week: OR: 0.63, 95% CI 0.48–0.82). Moreover, in comparison to the lack of consuming fried foods, its consumptions for less than once a week (OR: 1.93, 95% CI 1.14–3.27) and once or more per week (OR: 2.45, 95% CI 1.47–4.08) tend to cause a significant increase in stress symptoms. An extra adjustment for BMI did not alter the above results (OR: 1.84, 95% CI 1.07–3.16, OR: 2.47, 95% CI 1.46–4.18). We also found a significant relation between consumption of snacks for less than once (OR:1.61, 95% CI 1.22–2.13), once or more per week (OR:1.82, 95% CI 1.3–2.54) with stress features in compared with their non-consumption, which even after an auxiliary adjustment for BMI was significant (less than once per week: OR:1.41, 95% CI 1.05–1.88; once or more per week: OR:1.73, 95% CI 1.23–2.54). Meanwhile, the present study indicated an inverse association between consuming breakfast for more than once per week and lower chances of stress symptoms in comparison to skipping breakfast (OR: 0.6, 95% CI 0.39–0.92). This effect continues to exist even after the following adjustment for BMI (OR: 0.6, CI 0.39–0.92). Nevertheless, no further association was found between stress symptoms and other dietary habits included in the table.

**Table 5:** Multivariable-adjusted odds ratios (95% CI) for stress across different frequencies or servings of dietary habits

Dietary habits^a^	Stress			
	Multivariable adjusted^b^		Multivariable + BMI^c^	
	OR	95% CI	OR	95% CI
Sweetened drinks				
Not at all	Reference		Reference	
Lower than once per week	0.71*	0.54–0.94*	0.73*	0.55–0.97*
Once or more per week	0.67*	0.52–0.87*	0.63*	0.48–0.82*
Fast foods				
Not at all or few times per year	Reference		Reference	
1–3 times per month	1.08	0.83–1.41	1.14	0.86–1.49
Once or more per week	1.22	0.85–1.75	1.28	0.88–1.86
Canned foods				
Not at all or few times	Reference		Reference	
1–3 times per months	1.02	0.803–1.31	0.95	0.74–1.23
Once or more per week	1.22	0.82–1.82	1.05	0.69–1.59
Breakfast				
Not at all	Reference		Reference	
Once per week	1.11	0.59–2.06	1.03	0.54–1.96
More than once per week	0.60*	0.39–0.92*	0.601*	0.39–0.92*
Sugar cubes (one sugar cube contains almost 3 g sugar)				
Not at all	Reference		Reference	
1–2 cubes per day	0.78	0.56–1.07	0.78	0.56–1.08
More than 2 cubes per day	0.83	0.63–1.11	0.78	0.58–1.04
Fried foods				
Not at all	Reference		Reference	
Lower than once per week	1.93*	1.14–3.27*	1.84*	1.07–3.16*
Once or more per week	2.45*	1.47–4.08*	2.47*	1.46–4.18*
Snacks				
Not at all	Reference		Reference	
Lower than once per week	1.61*	1.22–2.13*	1.41*	1.05–1.88*
Once or more per week	1.82*	1.31–2.54*	1.73*	1.23–2.45*

*OR* odds ratio, *95% CI* 95% confidence interval

*Significance level was considered as *p* < 0.05

^a^Dietary habits for all items were presented as frequency of consumption except for sugar cubes which presented as serving of intake

^b^Adjusted for age (20–29, 30–39, 40–49, 50–59, 60–69 years), education level (Secondary school and lower, High school, Diploma and Graduate diploma, Bachelors, Masters, and PhD), physical activity level (low/medium/high), history of chronic diseases (hypertension, diabetes, cardiovascular disease, cancer, depression and dyslipidemia), and smoking (yes/no)

^c^Adjusted for age (20–29, 30–39, 40–49, 50–59, 60–69 years), education level (Secondary school and lower, High school, Diploma and Graduate diploma, Bachelors, Masters, and PhD), physical activity level (low/medium/high), history of chronic diseases (hypertension, diabetes, cardiovascular disease, cancer, depression, and dyslipidemia), smoking (yes/no), and BMI

## Discussion

The current study on a large population of Iranian adults found an inverse relationship between the consumption of sweetened drinks and psychological problems including depressive, anxiety, and stress symptoms, whereas intake of snacks was related to increased chances of psychological problems. A protective effect on stress features was also observed for eating breakfast. Moreover, consumptions of fast foods and fried foods were associated with increased chances of depressive and stress symptoms respectively.

Several surveys reported an inverse relationship between soft drinks’ intake and psychological problems present similar findings to the current research [14, 24, 33, 34].

In contrast to our research, some studies found that consumption of soft drinks was associated with increased risk of psychological problems in United States [19] and Australia [20]. Discrepancy between our findings and multiple studies could be attributed to differences in sample size, population characteristics, lifestyles, and dietary habits.

It has been found that drinks containing carbohydrates, especially high glycemic index (GI) ones, could cause rapid and immediate changes in serotonin levels and consequently improve mood or even relieve psychiatric disorders [35, 36]. Furthermore, we considered fruit juices, artificially or homemade sweetened beverages as sweetened drinks in our study. In Iran, homemade sweetened beverages are usually of plant origin that may contain beneficial compounds such as polyphenols. Evidence showed that polyphenols might have a preventive role against psychological problems such as depression due to having anti-inflammatory and anti-oxidative properties [37]. Therefore, an inverse association between sweetened drinks and chances of psychological problems in our research might be related to these issues.

Similar to the current survey, some studies reported that higher consumptions of energy dense foods such as cookies, fast foods, and snacks were related to greater risk of psychological problems [12, 13]. A significant association was also observed between greater fast foods intake and higher risk of depression in the surveys from United States of America (USA) [16] and China [17]. Furthermore, more palatable non-nutritious food consumption such as fried foods was associated with greater risk of stress [14].

Evidence has suggested that more fat ingestion might lead to a reduction in brain-derived neurotrophic factor which is a protein in charge of creating new neurons. This decrease might lead to a reduction in synaptic and cognitive function and neuronal growth that would implicate a development in psychological disorders [38]. Thus, a relationship between high consumption of fast foods, snacks, fried foods, and increased chances of psychological problems in our survey might be attributed to high-fat content of these foods.

Several studies reported a protective effect for consuming breakfast similar to our findings in adult population [39–41]. Moreover, the consequences of skipping breakfast were mostly considered as cognitive failures, lapses in attention, and concentration with of course mental distress in young adults [42].

The exact mechanism of the protective role of eating breakfast against psychological disorders is still unclear. However, the results of some surveys suggest that breakfast especially rich in carbohydrates can result in increased serotonin and decreased cortisol levels by altering the blood glucose [43, 44]. It has been also reported that eating breakfast regularly reduces stress levels and improves mental overall health by decreasing cortisol level [45]. Additionally, breakfast carbohydrate conversion into glucose which is necessary for the production of tryptophan, the precursor of serotonin, can be a potential explanation for regulating mood and cognitive function [46].

Our research had several strengths. To the best of our knowledge, it is the first population-based study that reports the association between dietary habits and psychological problems including depressive, anxiety, and stress symptoms among a large population in a Middle Eastern country. Furthermore, an extensive range of confounding factors that might influence psychological status was controlled. Nevertheless, the current study had some limitations. First, our survey might not accurately explain causality due to its cross-sectional design. The measurement error was also another limitation which is an identified feature of any dietary evaluation method. Third, we could not control the impact of all the confounders due to unknown or unmeasured factors. Moreover, psychological assessment was performed by a self-rated psychological scale (DASS-21) which only evaluated psychological problems symptoms in a recent small period. Therefore, the DASS-21 might not be an appropriate tool for making a precise psychological diagnosis.

## Conclusion

In conclusion, the current research indicated an inverse association between intakes of sweetened drinks and depressive, anxiety, and stress symptoms, whereas snacks consumption was related to increased chances of them. Furthermore, intake of fast foods and fried foods were associated with increased chances of depressive and stress features, respectively. A protective effect was also found for eating breakfast on stress symptoms. Further surveys, especially population-based cohort studies, are recommended to provide more conclusive evidence for explaining the relationship between dietary habits and psychological problems.

## Data Availability

Not applicable.

## Abbreviations

OR: Odds ratio
CI: Confidence interval
CVD: Cardiovascular disease
YaHS: Yazd Health Study
BMI: Body mass index
DASS 21: Depression, anxiety, and stress scale questionnaire 21
IPAQ: International Physical Activity Questionnaire
GI: Glycemic index
USA: United States of America
